# Chloroplast genome characteristics of *Corylopsis microcarpa* H.T. Chang (Hamamelidaceae)

**DOI:** 10.1080/23802359.2022.2152643

**Published:** 2022-12-09

**Authors:** Jinsen Lu, Kai Xu, Xiaohong Qiu, Mu Liu

**Affiliations:** a College of Landscape Architecture and Arts, Jiangxi Agricultural University, Nanchang, China; b College of Forestry, Jiangxi Agricultural University, Nanchang, China; c Garden Bureau of Yushan County, Yushan, China

**Keywords:** *Corylopsis microcarpa*, endemic species, phylogenetic relationship, chloroplast genome

## Abstract

*Corylopsis microcarpa* H.T. Chang 1960 is a relict species from China. The chloroplast genome of *C. microcarpa* is 159,438 bp in size and shows typical quadripartite structure, which includes a pair of inverted repeat regions (26,280 bp), a large single-copy region (88,185 bp), and a small single-copy region (18,693 bp). The whole chloroplast genome encodes 114 unique genes, including 80 protein-code genes, 30 transfer RNA (tRNA) genes, and four ribosomal RNA (rRNA) genes. Ninety-one SSRs were identified. The phylogenetic analysis revealed *C. microcarpa* diverged early in *Corylopsis*.

*Corylopsis microcarpa* H.T. Chang 1960, belonging to Hamamelidaceae, is mainly distributed in forests or mountains of southwestern and northern regions in China (Morley and Chao 1977). *C. microcarpa* is a relict species, and the number of its wild populations have declined as a result of habitat destruction and fragmentation (Qin et al. 2017). In the present study, we sequenced the complete chloroplast genome of *C. microcarpa* and performed the phylogenetic analysis with other related species within the family of Hamamelidaceae. The chloroplast genomic resources are important for assessing population genetics, species identification (Dong, Sun, et al. 2021) and phylogenetic analysis (Dong, Liu, et al. 2021; Li L et al. 2021; Dong, Liu, et al. 2022).

Fresh and young leaves of *C. microcarpa* were collected and identified by Dr. Mu Liu from Wenxian, Gansu, China (32°44′38″∼105°14′31″). The voucher specimen was deposited at the herbarium of Jiangxi Agricultural University under the voucher number of LM850241 (http://english.jxau.edu.cn/, Mu Liu, aawolongaa@163.com). Total DNA was extracted using a modified CTAB DNA extraction protocol (Li J et al. 2013). We used genome skimming method to sequence the chloroplast genome of *C. microcarpa* (Dong, Sun, et al. 2021; Dong, Li, et al. 2022). Total DNA was fragmented to construct a shotgun library and sequenced on the Illumina HiSeq platform. Approximately 5 Gb of data was generated. Raw data was cleaned and filtered using Trimmomatic (Bolger et al. 2014) and complete chloroplast genome was assembled utilizing GetOrganelle (Jin et al. 2019), with a kmer length of 95. Chloroplast genome of *C. microcarpa* was annotated using Perl script Plann (Huang and Cronk 2015).

The *C. microcarpa* chloroplast genome is 159,438 bp in length with standard quadripartite structure. The complete chloroplast genome contains a pair of IRs with the length of 26,280 bp separated by a large single copy with the length of 88,185 bp and a small single copy with the length of 18,693 bp. The overall GC content of the genome is 38%. The *C. microcarpa* chloroplast genome encodes 114 unique genes, including 80 protein-code genes, 30 transfer RNA (tRNA) genes, and four ribosomal RNA (rRNA) genes. Microsatellite sites were identified using Genome-wide Microsatellite Analyzing Tool Package (GMATA) software (Wang and Wang 2016). The minimum number of repeats was set to ten for mono, five for di-, four for tri-, and three each for tetra-, penta-, and hexanucleotide SSRs. The total number of SSRs identified in *C. microcarpa* chloroplast genome was 91. These SSRs included 70 mononucleotide, 9 dinucleotide, 3 trinucleotide, 6 tetranucleotide and 3 pentanucleotide SSRs.

To resolve the phylogenetic position of *C. microcarpa* in Hamamelidaceae, a total of 29 complete chloroplast genome sequences were downloaded from the GenBank database. Chloroplast genome sequences were aligned using MAFFT (Katoh and Standley 2013) and the ambiguous alignment regions were trimmed using TrimAl (Capella-Gutiérrez et al. 2009). ML analysis in RAxML-NG (Kozlov et al. 2019) was used to infer phylogenetic relationships. The best-fit model was selected by ModelFinder (Kalyaanamoorthy et al. 2017). The phylogenetic analysis revealed all the nodes in the phylogenetic tree had high bootstrap support values (Figure 1). Subfamily Disanthoideae was sister to Hamamelidoideae. The seven *Corylopsis* species formed a clade and were sister to *Loropetalum. C. microcarpa* was sister to a clade including *C. coreana, C. spic*ata, C. spicata, C. glandulifera, C. sinensis and C. velutina with high support value (BS = 100).

**Figure 1.**
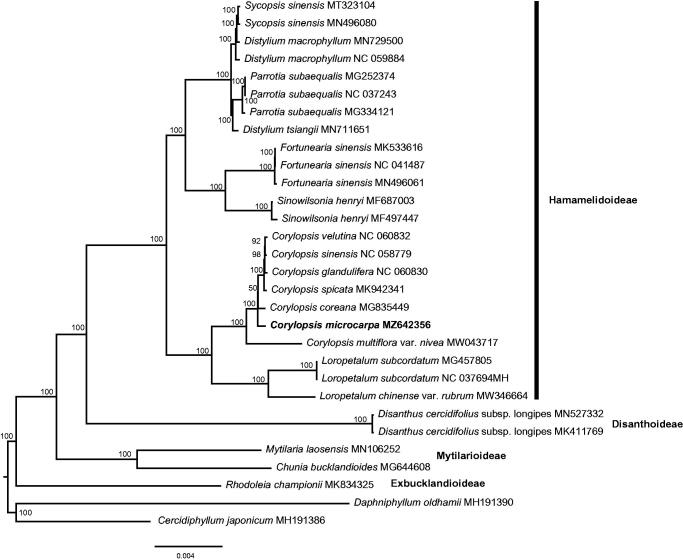
Phylogenetic tree of Hamamelidaceae based on 29 complete chloroplast genome sequences. ML topology shown with ML bootstrap support value at each node.

## Data Availability

The genome sequence data that support the findings of this study are openly available in GenBank of NCBI at (https://www.ncbi.nlm.nih.gov/) under the accession no. MZ642356. The associated BioProject, SRA, and Bio-Sample numbers are PRJNA749546, SRX11547405, and SAMN20375765, respectively.
